# Dual processing streams in chemosensory perception

**DOI:** 10.3389/fnhum.2012.00288

**Published:** 2012-10-19

**Authors:** Johannes Frasnelli, Johan N. Lundström, Veronika Schöpf, Simona Negoias, Thomas Hummel, Franco Lepore

**Affiliations:** ^1^Centre de Recherche en Neuropsychologie et Cognition, Université de MontréalMontréal, QC, Canada; ^2^Department of ENT-Medicine, Technical University of DresdenDresden, Germany; ^3^Cognitive Neuroimaging Laboratory, Monell Chemical Senses CenterPhiladelphia, PA, USA; ^4^Department of Clinical Neuroscience, Karolinska InstituteStockholm, Sweden; ^5^Department of Radiology, Division of Neuro- and Musculoskeletal Radiology, Medical University ViennaVienna, Austria

**Keywords:** olfaction, trigeminal system, independent component analysis, general linear model, ventral, dorsal

## Abstract

Higher order sensory processing follows a general subdivision into a ventral and a dorsal stream for visual, auditory, and tactile information. Object identification is processed in temporal structures (ventral stream), whereas object localization leads to activation of parietal structures (dorsal stream). To examine whether the chemical senses demonstrate a similar dissociation, we investigated odor identification and odor localization in 16 healthy young subjects using functional MRI. We used two odors—(1) eucalyptol; (2) a mixture of phenylethanol and carbon dioxide)—which were delivered to only one nostril. During odor identification subjects had to recognize the odor; during odor localization they had to detect the stimulated nostril. We used general linear model (GLM) as a classical method as well as independent component analysis (ICA) in order to investigate a possible neuroanatomical dissociation between both tasks. Both methods showed differences between tasks—confirming a dual processing stream in the chemical senses—but revealed complementary results. Specifically, GLM identified the left intraparietal sulcus and the right superior frontal sulcus to be more activated when subjects were localizing the odorants. For the same task, ICA identified a significant cluster in the left parietal lobe (paracentral lobule) but also in the right hippocampus. While GLM did not find significant activations for odor identification, ICA revealed two clusters (in the left central fissure and the left superior frontal gyrus) for this task. These data demonstrate that higher order chemosensory processing shares the general subdivision into a ventral and a dorsal processing stream with other sensory systems and suggest that this is a global principle, independent of sensory channels.

## Introduction

Over the last 20 years, neuroimaging methods such as positron emission tomography (PET) and functional magnetic resonance imaging (fMRI) have allowed for the investigation of brain regions involved in olfactory processing. Since Zatorre and colleague's seminal paper, in which they localized olfactory information processing to piriform and orbitofrontal cortex (Zatorre et al., 1992), researchers have investigated cerebral areas involved in different olfactory tasks. The olfactory system has been suggested to be dependent on concurrent parallel and hierarchial pathways. According to this model, olfactory stimulation always leads to activation of basic olfactory processing areas, such as the piriform cortex, amygdala, orbitofrontal cortex and insula, independent of the task. Higher order brain structures (e.g., prefrontal cortex) are thereafter activated dependent on the specific task (e.g., olfactory memory) (Savic et al., 2000). For example, by presenting two concentrations (low and high) of each a pleasant and an unpleasant odor, Anderson et al. investigated cortical representation of odor valence and intensity. They observed an intensity-dependent activation of the amygdala disassociated from odor valence. Regions of the orbitofrontal cortex, in contrast, were differentially activated by odor valence where odor intensity had no effect (Anderson et al., 2003). However, these results were later suggested to be paradigm rather than intensity-dependent (Winston et al., 2005). Later a study with a similarly elegant design investigated the effect of chemical structure and quality using cross adaptation. Here, anterior portions of the piriform cortex were demonstrated to encode information regarding chemical structure, whereas posterior parts of the same area responded independent of chemical structure, but differentially to odor quality (Gottfried et al., 2006). These studies provide evidence that the olfactory system is indeed organized both hierarchically and topographically. This is in analogy to the other sensory systems, such as tonotopy in audition, with high and low frequencies being represented in medial and lateral portions, respectively, of the Heschl gyrus (Schonwiesner et al., 2002) or retinotopy in vision, where neighboring retinal areas project to adjacent cortical areas in the occipital cortex (Grill-Spector and Malach, 2004).

Analogies between the senses may extend beyond hierarchical and topographical organization with dual processing streams being a possible candidate. Two separate processing streams, the dorsal and ventral stream, were first described within the visual system of the monkey (Mishkin et al., 1983). The dorsal stream, extending from visual cortex to posterior parietal cortex, has a role in spatial perception (i.e., localization of an object in space, “where is the object?”), whereas the ventral stream to the temporal cortex processes object perception (i.e., identification of an object, “what is the object?”). This model of separation of processing according to stimulus characteristics has subsequently been confirmed also within the human visual (Haxby et al., 1991), auditory (Rauschecker and Tian, 2000), and somatosensory systems (Reed et al., 2005), as well as in multisensory integration (Renier et al., 2009).

Whether a dual processing stream also exists within the chemical senses is not known. Several lines of evidence do, however, indicate that a separation according to stimulus characteristics exists. The intranasal chemical systems are able to extract information related to both object localization, a ventral stream associated process, as well as object identification, a dorsal stream associated process. The main noticeable difference to our visual and auditory senses is that these abilities—especially object localization—are dependent on associative processing in two separate senses, the olfactory and trigeminal sense. While object identification is evident for olfaction (Doty et al., 1984), localization of odorous objects seems to be more difficult, if not impossible, for humans based on the sense of smell solely. When directional smelling, i.e., the ability to localize odors in space, is assessed in humans, researchers achieve a maximal concentration gradient between both nostrils by stimulating only one nostril with an odor; the other nostril receives only air. Even in this extreme case, subjects were not able to correctly localize the stimulated nostril unless the odor additionally stimulates the intranasal trigeminal system (von Skramlik, 1924; Schneider and Schmidt, 1967; Wysocki et al., 2003; Frasnelli et al., 2009, 2010; Kleemann et al., 2009; Wise et al., 2012); these odors are called mixed olfactory trigeminal stimuli as opposed to pure odors, which stimulate the sense of smell exclusively (Kobal et al., 1989). Nonetheless, studies have claimed that localization of pure odors is possible for humans (von Békésy, 1964; Porter et al., 2005), however, they used odors which under certain circumstances are known to stimulate the trigeminal nerve (Frasnelli et al., 2011). In fact, the vast majority, if not all, odors stimulate the trigeminal nerve, at least in higher concentrations (Doty et al., 1978; Frasnelli et al., 2011) rendering a pure odor sensation a very rare event (Wise et al., 2012).

Nasal stimulation with a pure odorant (phenyl ethanol), a pure trigeminal stimulus (carbon dioxide), and a mixture thereof were recently studied in more detail (Boyle et al., 2007). The pure odor activated brain areas classically considered to be olfactory (piriform cortex—PIR, and orbitofrontal cortex—OFC). The pure trigeminal stimulus, in turn, activated the somatosensory brain areas (thalamus, postcentral gyrus) as well as those aforementioned olfactory related areas. The mixture of both stimuli, however, activated additional brain areas than the sum of the activations to the individual components. Specifically, the mixture activated chemosensory processing areas (PIR, OFC) and multisensory integration areas located in the parietal lobe (such as the intraparietal sulcus—IPS) and the temporal lobe (such as the superior temporal sulcus—STS) more than the individual components (Boyle et al., 2007).

Mixed olfactory-trigeminal stimuli which are both identifiable (Doty et al., 1984; Laska et al., 1997) and localizable (Kobal et al., 1989; Frasnelli et al., 2009, 2011) are therefore good stimulus candidates if one aims to investigate whether a dual processing stream using a ventral and a dorsal pathway exists in the chemical senses, akin our other senses.

The literature provides us with several brain regions in which multisensory integration takes place on a cortical level. Some studies compared superadditive effects of multimodal compared to unimodal stimulation, e.g., for auditory and visual stimuli (Calvert et al., 2001) or for olfactory and trigeminal stimuli (Boyle et al., 2007). The resulting activation maps of both studies overlapped partially, and exhibited superadditive effects for the insula, IPS, STS, as well as frontal regions (middle and superior frontal gyrus). Some of these multisensory integration areas were activated in a task specific manner in another study which used auditory and vibrotactile multimodal stimuli. Here, the task of localizing the stimuli activated parietal cortex (left and right inferior parietal lobule—IPL, right precuneus, superior parietal lobule—SPL), whereas identifying the stimuli activated bilateral insula, and right inferior frontal gyrus (Renier et al., 2009). These multisensory integration areas are prime candidates to serve as nodes also within a chemosensory ventral and a dorsal stream.

The aim of this study was to determine the existence of a separation into a ventral and dorsal stream for chemosensory processing. In contrast to earlier studies, which showed dual streams for monomodal processing, we aimed to investigate this question by using stimuli which stimulated separate sensory systems, i.e., the olfactory system and the trigeminal system. Despite the fact that both sensory systems exhibit distinct peripheral pathways—the olfactory nerve and bulb as well as piriform cortex for the olfactory system, the trigeminal nerve and ganglion, thalamic rely for the trigeminal system—they share important central processing areas such as the orbitofrontal cortex and the insula (Boyle et al., 2007; Albrecht et al., 2010). These brain areas can therefore be considered chemosensory processing areas (Albrecht et al., 2010), in line with the notion of a unique flavor sense (Auvray and Spence, 2008) integrating inputs from different sensory channels to one single percept.

In this study we used both exploratory and model driven fMRI analyses. To this extent, we performed a standard regression based fMRI analysis based on the general linear model (GLM) and compared the results to the fully exploratory method based on independent component analysis (ICA). Chemosensory experiments are susceptible to factors such as movement due to the very nature of the stimulus. Although motion parameters of the subject can be included as nuisance regressors in the GLM analysis, this reduces motion effects particularly in event-related designs (Birn et al., 1999), but BOLD sensitivity is substantially reduced even if a moderate correlation between motion and task is present (Johnstone et al., 2006). These time-locked effects can lead to false positive results in a GLM (Hajnal et al., 1994). We therefore additionally performed an analysis based on spatial ICA, which is able to isolate activation in data based on spatial independence rather than temporal similarity between stimulus and response adding a beneficial factor to the analysis in this study.

## Materials and methods

### Subjects

We included 16 healthy, young participants (12 women, mean age: 24; 20–29 years) in this study. The study was conducted at the University of Dresden Medical School, according to the Declaration of Helsinki and all subjects gave written informed consent prior to the study. It was approved by the local Ethics Committee (EK number 185062009).

### Behavioral testing

Before the fMRI session, we assessed subjects' olfactory abilities and trigeminal chemoreception. Subjects' ability to identify odors was determined by means of the Sniffin' Sticks identification test kit (Kobal et al., 2000). In this test, subjects are presented with 12 pen-like odor dispensing devices. Their task was to choose the right descriptor from a list of four for each odor. We counted the number of correct responses. Further, we assessed subjects' ability to localize odors by means of the odor lateralization test for eucalyptol (Hummel et al., 2003). We stimulated subjects with a device which allows the delivery of predefined volumes of air to both nostrils simultaneously. In this test, subjects were stimulated with odorized air to one nostril and odorless air to the other; their task was to detect the side of odor stimulation. We used neat eucalyptol as the odor stimulus. The task was repeated 40 times with each trial separated by 40 s. We counted the number of correct localizations. We only included participants with a normal ability to identify odors [i.e., who were able to identify more than 10 out of 12 sticks (Hummel et al., 2001)] and the ability to localize odors above chance [more than 25 out of 40 (Frasnelli et al., 2008)].

### Chemosensory stimuli

We used 20% of eucalyptol saturated air (eucalyptus odor) and a mix of 20% of phenyl ethyl alcohol saturated air (rose odor) with 60% carbon dioxide (CO_2_) (Boyle et al., 2007) as bimodal odors; both are known to activate the olfactory and the intranasal trigeminal system (Hummel et al., 2003; Boyle et al., 2007). The reasons why we decided to use a mixture of phenyl ethanol and carbon dioxide instead of a monomolecular substance are two-fold. First, phenyl ethanol is a pure odorant and therefore very difficult to be localized by humans (Frasnelli et al., 2009); carbon dioxide is virtually odorless and therefore very difficult to identify; the mixture of both, however, is both localizable and identifyable (Boyle et al., 2007). Second, it is difficult to match a monomolecular substance with regards to olfactory and trigeminal intensity. By using a mixture, we could adjust both trigeminal and olfactory intensity (by changing the concentrations of phenyl ethanol and CO_2_ separately) to match eucalyptol's in pilote experiments. Stimuli were therefore isointense on both, the olfactory and the trigeminal dimensions. Subjects were familiarized with both odors and could easily distinguish them.

### Odor presentation

Odor stimuli were applied by means of a computer-controlled air-dilution olfactometer (OM6b; Burghart, Wedel, Germany). This stimulator allows application of rectangular-shaped chemical stimuli with controlled stimulus onset. Mechanical stimulation is avoided by embedding stimuli into a constant flow of odorless, humidified air of controlled temperature (80% relative humidity, total flow 8 L/min, 36°C) (Kobal, 1981). Thus, throughout the experiment, the subjects received humidified, warm air to their nostril. During stimulation an odor was embedded into this constant airflow. The olfactometer allows for stimulation of each nostril separately. Subjects were instructed to breathe through their mouth to avoid potential sniff-related activity.

### Testing paradigm

We used a block design for stimulation. During the entire fMRI session, subjects focussed on a black cross on a screen. Nine seconds before the “on-period”, the cross switched to one of two questions [task; either “where?” (German: “wo?”) or “what?” (“was?”)]. The order of the questions was pseudo-randomized and counterbalanced. The text stayed on the screen for 5 s after which it switched back to the black cross. Four seconds later, the “on”-period begun during which odor stimuli were delivered five times, each 400 ms long, every 4 s. The chemosensory stimuli were either eucalyptol or the PEA/CO_2_ mixture and delivered either to the left or the right nostril (all stimuli pseudorandomized and counterbalanced). After each stimulus, subjects responded to the task by pressing one of two buttons with the index of their right hand. Specifically, during the localization task they had to indicate whether their left or their right nostril was stimulated and during the identification task, they had to indicate whether they received eucalyptol or the PEA/CO_2_ mix. The “on”-period was followed by a 30 s “off”-period, during which subjects received odorless air (AIR). During one run, we delivered 10 “on”- and 10 “off”-periods; subjects were tested in two runs. For data analysis we classified volumes during the “on”-periods as “where” or “what” conditions, whereas the “off”-periods were classified as “baseline” condition.

### Image acquisition

The study was performed using a 1.5 MRI scanner (Sonata; Siemens, Erlangen, Germany). For anatomical overlays, a T1-weighted (turboflash sequence) axial scan with 224 slices, voxel size of 1.6 × 1.1 × 1.5 mm, a repetition time (TR) of 3000 ms, echo time (TE) of 3.93 ms, and 2 averages (2130/3.93/2) was acquired. Functional data acquisition was performed in the axial plane (oriented parallel to the planum sphenoidale to minimize artifacts) using a multislice spin-echo echo-planar imaging sequence. Scan parameters included a 64 × 64 matrix, voxel size of 3 × 3 × 3.75 mm, TR of 3000 ms, and a TE of 35 ms. A total of 207 images were acquired at each of 24 slice locations per run over the course of a total functional acquisition session of approximately 10 min in length. The three imaging conditions consisted of (1) subjects identifying a chemosensory stimulus (“what”), (2) subjects localizing a chemosensory stimulus (“where”), and (3) chemosensory-free low-level baseline.

### Data analysis

The functional MRI data was analyzed by means of SPM8 (Wellcome Trust, http://www.fil.ion.ucl.ac.uk/spm/software/spm8/) implemented in Matlab (Mathworks Inc., Natick, MS). Functional data were registered, motion-corrected, and resliced using SPM8 preprocessing procedures. Mean functional images were coregistered to the anatomical T1 volume. We then performed the analysis on spatially normalized stereotactically transformed into ICBM152-space and smoothed images (8 mm full width at half maximum Gaussian kernel).

#### General linear model—GLM

We calculated a second level analysis contrasting images using a paired sample *t*-test to highlight the difference between conditions (what and where vs. baseline; what vs. where; where vs. what). We corrected for whole brain family-wise error (FWE) thresholding at *p* < 0.05 If this analysis yielded no significant result, we lowered the criterion to *p* < 0.001 uncorrected (indicated as “uncorrected”) and then only reported areas where we had a strong a-priori hypothesis of result based on the existing literature. Moreover, in order to minimize the potential for false positive findings, indeed a worry when reporting uncorrected results, we set the cluster criterion to 10 voxels to only detect areas of extended neural activity, thus lowering the possibly of results based on random fluctuations.

#### Independent component analysis—ICA

Functional data sets were post-processed using probabilistic independent component analysis (P-ICA) (Beckmann and Smith, 2004) as implemented in MELODIC (Multivariate Exploratory Linear Decomposition into Independent Components) version 3.10, a part of FSL (FMRIB's Software Library, www.fmrib.ox.ac.uk/fsl). The optimum number of components to be estimated was 29, determined using the implemented criterion Minimum Description Length (MDL) (Rissanen, 1978). Regression was used by utilizing the first two stages of the dual regression approach version v0.5, a part of FSL (Filippini et al., 2009) to obtain single-subject specific component maps and time courses. For each individual subject temporal correlations revealed the single-subject independent component (IC) with the best fit for both conditions (condition 1: “what,” condition 2: “where”) using Matlab (Matlab 7.8, Release 2009a). Corresponding spatial IC maps for every subject and both conditions were then exported to SPM8 for statistical testing and visualization. For second-level analysis, two separate *t*-tests were performed for both conditions (*p* < 0.05, FWE corrected). Again, the cluster threshold was set at 10 voxels.

## Results

### General linear model (GLM)

In order to first verify that our imaging paradigm reliably activated chemosensory processing areas, we initially assessed the main effect of odor stimulation by comparing both odor conditions against no odor condition (where + what vs. no-odor baseline). We observed activations areas commonly associated with chemosensory processing, such as left and right insula, the right OFC, as well as multisensory integration centers such as the right inferior parietal lobule and the left supramarginal gyrus (see Table 1).

**Table 1:** **Brain activation due to stimulation with eucalyptus and a phenyl ethanol/ CO_2_ mixture: comparison of both tasks vs. baseline [contrast (where & what) vs. baseline]**.

**Area**	***x***	***y***	***z***	***T***	**Voxels**
Right insula	54	14	4	10.1	260
Left insula	−42	14	1	6.8	42
Right lateral OFC	45	44	−5	8.5	38
Right inferior parietal lobule	51	−37	49	6.9	20
Right middle frontal G	42	41	19	7.1	23

p < 0.05, corrected, extent threshold 10 voxels.

To verify task specific brain activations, we compared the two stimulation conditions to each other. When contrasting odor localization against odor identification (where vs. what), we observed activations of a cluster in the left intraparietal sulcus, and one in the right superior frontal sulcus (Table 2).

**Table 2:** **Brain activation due to stimulation with eucalyptus and a phenyl ethanol/CO_2_ mixture: comparison of between odor localization vs. odor identification (contrast where—what)**.

**Area**	***x***	***y***	***z***	***T***	**Voxels**
Left intraparietal sulcus	−36	−43	31	4.7	10
Right superior frontal sulcus	21	20	34	4.3	10

p < 0.001, uncorrected, extent threshold 10 voxels.

The opposite contrast (what vs. where) did not reveal any significant activation above threshold criteria.

### Independent component analysis (ICA)

We used ICA to obtain specific component maps for individual subjects. We then extracted, for each subject, the component with the best fit to the time course of each condition. Information on correlation coefficients for individual components in both tasks is outlined in Table 3.

**Table 3:** **Correlation between independent component and task (left: “where“; right: “what”) per subject**.

**Subject**	**“where”**				**“what”**			
	**max cc**	**IC#**	**mean abs cc**	**SD abs cc**	**max cc**	**IC#**	**mean abs cc**	**SD abs cc**
1	0.03	4	0.06	0.04	0.02	4	0.06	0.06
2	0.21	14	0.07	0.06	0.21	16	0.09	0.05
3	0.09	5	0.09	0.05	0.13	2	0.07	0.05
4	0.07	11	0.05	0.03	0.13	10	0.08	0.07
5	0.05	16	0.03	0.02	0.16	16	0.04	0.04
6	0.05	7	0.04	0.03	0.11	2	0.06	0.03
7	0.13	2	0.11	0.08	0.10	13	0.08	0.06
8	0.06	4	0.05	0.03	0.09	4	0.05	0.03
9	0.20	1	0.10	0.07	0.31	9	0.14	0.08
10	0.10	14	0.07	0.04	0.23	14	0.09	0.06
11	0.11	14	0.04	0.04	0.12	16	0.05	0.04
12	0.08	7	0.05	0.03	0.16	6	0.05	0.04
13	0.08	7	0.07	0.05	0.16	16	0.06	0.05
14	0.21	4	0.10	0.08	0.13	13	0.07	0.06
15	0.09	7	0.05	0.05	0.17	16	0.07	0.05
16	0.03	16	0.10	0.08	0.08	14	0.11	0.08

Subject, consecutive subject ID; max cc, maximal correlation coefficients; IC#, number independent component corresponding to max cc; mean abs cc, mean of absolute values of correlation coefficients; SD abs cc, standard deviation of absolute values of correlation coefficients.

The resulting statistical maps were submitted to a subsequent second level analysis where the component for odor identification (“what”) revealed two significant clusters within an area of the left central fissure and the left superior frontal gyrus (Table 4).

**Table 4:** **Brain activation due to stimulation with eucalyptus and a phenyl ethanol/CO_2_ mixture: independent component analysis: component fitting best for odor identification [ICA (what)]**.

**Area**	***x***	***y***	***z***	***T***	**Voxels**
Left central fissure	−24	−31	52	15.9	19
Left superior frontal gyrus	−24	−16	40	5.92	20

p < 0.05, corrected, extent threshold 10 voxels.

For odor localization (“where”), we detected two clusters above set criterion, one located in the right hippocampal region and another in left paracentral lobule (Table 5).

**Table 5:** **Brain activation due to stimulation with eucalyptus and a phenyl ethanol/CO_2_ mixture: independent component analysis: component fitting best for odor localization [ICA (where)]**.

**Area**	***x***	***y***	***z***	***T***	**Voxels**
Right hippocampus	30	−46	4	6.73	19
Left paracentral lobule	−3	−31	55	5.21	11

p < 0.05, corrected, extent threshold 10 voxels.

**Figure 1:**
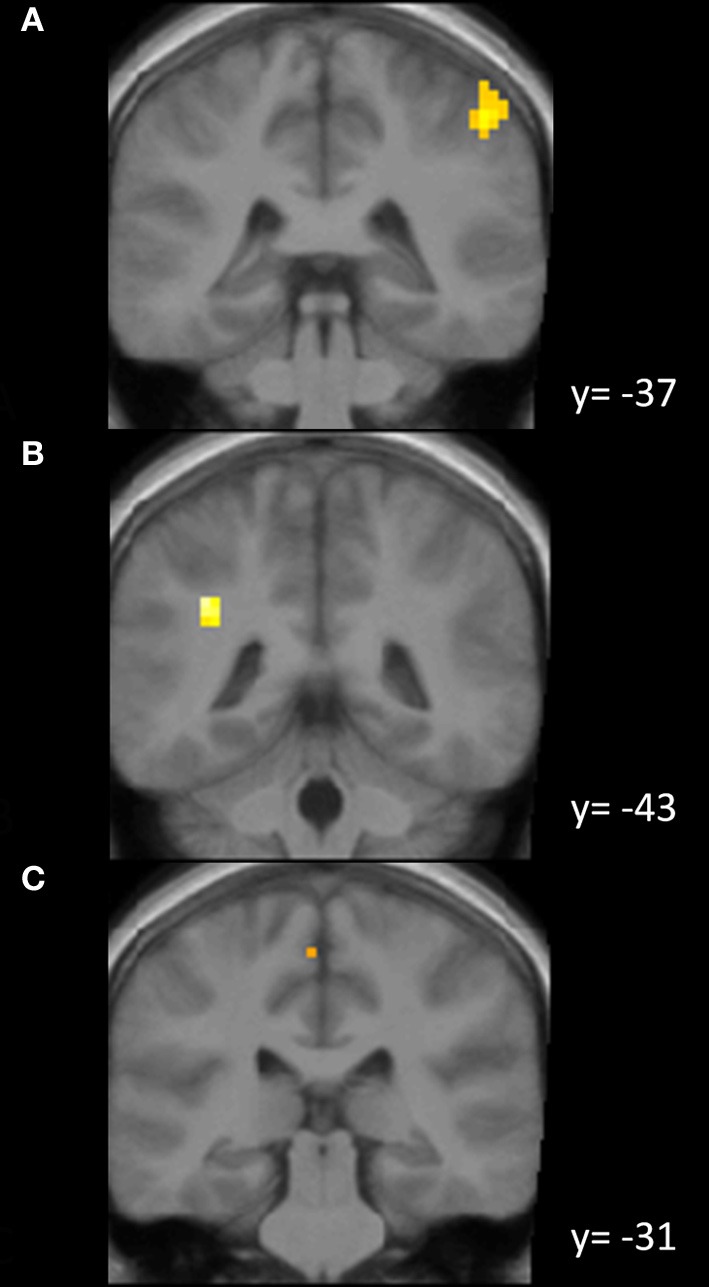
**Activations in the parietal lobe. (A)** Activation in the right inferior parietal lobule due to odorant perception [GLM—contrast: (odor identification and odor localization) vs. baseline; *p* < 0.05 (corrected)]; **(B)** activation in the left intraparietal sulcus due to odor localization [GLM—contrast: odor localization vs. odor identification; *p* < 0.001 (uncorrected)]; **(C)** activation in the left paracentral lobule due to odor localization [ICA—component fitting best for odor localization; *p* < 0.05 (corrected)].

In Figure 1 we provide an overview of activations in the parietal cortex obtained in different conditions (Figure 1).

## Discussion

In this study we examined whether processing of chemosensory information displays a subdivision into localization and identification following the notion of a dual stream demonstrated for other senses (Mishkin et al., 1983). We investigated localization and identification of mixed trigeminal-olfactory objects and observed that subjects activated distinct brain regions. Specifically, when subjects localized unilaterally presented mixed olfactory-trigeminal stimuli, regions in the left intraparietal sulcus and the right superior frontal sulcus were activated to a higher degree than if they were identifiying the same stimuli. Further, an ICA allowed us to extract task specific networks for each odor localization and odor identification. For odor localization, the network revealed two clusters, one in the right hippocampus, and one in the left paracentral lobule. For odor identification, also two clusters could be observed, one located around the left central fissure, the other one in the left superior frontal gyrus.

### Dual chemosensory processing streams

When subjects were localizing the odorous objects, both means of analysing the neuroimaging data identified significant activation of the left posterior parietal lobe, in addition to its general activation independent of the task. These observations fit well with the literature where object localization consistently activates posterior parietal regions. For instance, in analogy to the findings in non-human primates (Mishkin et al., 1983) a visual spatial localization task led to activation in the lateral superior parietal cortex (Haxby et al., 1991). In the auditory system, object localization activated a dorsal stream from the caudal primary auditory cortex to the inferior parietal cortex (somewhat lower than for visual stimuli) to middle and inferior frontal gyri, whereas anterior primary auditory cortex to posterior frontal and orbitofrontal regions formed the ventral stream for object identification (Rauschecker and Tian, 2000; Maeder et al., 2001).

Different subregions of the parietal lobe play particular roles in the dual stream dichotomy. SPL and IPS, whose activation is often associated with activation of the dorsoloateral frontal lobe, are part of the dorsal frontoparietal system for directing spatial attention. IPL on the other hand, is activated, together with more ventral frontal regions when individuals perform non-spatial tasks. Thus, there is a gradient from more spatial tasks in SPL to less spatial tasks in IPL, with the IPL's suggested role to sustain attention over time (Husain and Nachev, 2007). The data obtained within the present study corresponds closely with these earlier reports, especially with regards the parietal lobe: unilateral chemosensory stimuli triggered activation of right IPL independent of the task subjects performed, indicating multisensory integration. Localization of these stimuli, however, led to a significantly stronger activation of the left IPS than what we observed for stimulus identification. Therefore, the cortex in and around the IPS is part of a dorsal stream responsible for object localization in different sensory systems, including the chemical senses. Activation of this particular brain region was observed when subject localized monomodal stimuli, such as visual (Haxby et al., 1991), auditory (Rauschecker and Tian, 2000; Maeder et al., 2001), and somatosensory (Reed et al., 2005) ones as well as multimodal stimuli such as audio-somatosensory (Renier et al., 2009) and olfactory-trigeminal ones (present study). It is commonly activated with mixed olfactory trigeminal stimuli (Boyle et al., 2007; Lombion et al., 2009).

**Figure 2:**
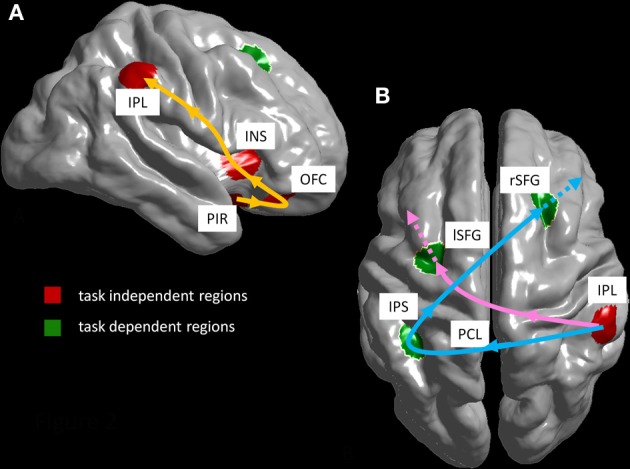
**Suggested pathways: (A) task-independent pathway (regions in red; orange arrow) from piriform cortex (PIR) via orbitofrontal cortex (OFC) and insula (INS) to right inferior parietal lobule (IPL). (B)** Task-dependent pathway (regions in green): localization pathway (blue arrow) from right inferior parietal lobule (IPL) via left intraparietal sulcus (IPS) and left paracentral lobule (PCL) to right superior frontal gyrus (rSFG); odorant identification pathway (pink arrow) from IPL to left superior frontal gyrus (lSFG).

Next to the parietal activations, we also observed activations in the frontal lobe. First, object localization led to a significant activation of the right superior frontal sulcus, as shown by the GLM contrast “where” vs. “what.” Second, the ICA demonstrated odor object identification to be associated with the left superior frontal gyrus. In addition to these hemispheric differences, the latter activation was located more posteriorily than the former. It has been demonstrated that there are distinct working memory systems for spatial and verbal information predominantly located in the (dorsolateral) prefrontal cortex of both hemispheres. The right hemisphere stores and maintains information on spatial features, whereas the left hemispheres does the same for verbal and object identity information (Smith and Jonides, 1997; Belger et al., 1998). This appears to be modality independent, as both visual and tactile working memory evoked similar frontoparietal networks including the posterior parietal cortex and the dorsolateral prefrontal cortex, with a leftwards tendency for object discrimination (Ricciardi et al., 2006). In Figure 2 we highlight how our results fit into the same framework. After activation of chemosensory regions common to both tasks, both tasks activated a parieto-frontal network, from the posterior parietal cortex to prefrontal areas, with activation of a left sided and a right sided frontal area for object identification and localization, respectively (Figure 2).

ICA revealed a puzzling finding in the activation of the right posterior hippocampus when subjects were localizing odors. Hippocampal activation is usually linked to spatial navigation and episodic memory (Igloi et al., 2010). Localizing odorants to the left or right nostril is clearly a spatial task; however, our paradigm did not explicitly involve a mnesic component. We know that hippocampus also stores serial working memory of spatial locations even in the encoding phase (Toepper et al., 2010). Importantly, this is true even in implicit conditions: hippocampal activation can be observed when subjects learn the temporal structure of sequences even without any conscious sequence knowledge (Schendan et al., 2003). In our paradigm, we presented our subjects with a series of spatial locations. Therefore, implicit spatial sequence learning may therefore explain the hippocampal activity we observed.

There has been a prior attempt to investigate the dissociation between object localization and identification in the chemical senses (Porter et al., 2005). Here, subjects smelled four odorants. Similarly to our study, odors were delivered monorhinally, and subjects were asked to either identify or localize the odors. Although the main focus of the study was to investigate nostril specific receptive fields within the piriform cortex, the authors also compared brain activations between both tasks. They did indeed observe dissociations between tasks which differentially activated three specific brain areas: odor identification activated the occipital gyrus and the paracentral lobule to a larger extent than odor localization; odor localization, in turn, activated the superior temporal gyrus more than odor identification. These findings contradict the existing literature where activation of different regions of the occipital cortex has been reported mainly for visual stimuli, during object identification [e.g., the occipitotemporal junction for face recognition (Haxby et al., 1991)] rather than for object localization. On the other hand, temporal areas have been associated with object identification rather than object localization (Mishkin et al., 1983), with the exception of sound localization (Maeder et al., 2001). Further, the existing literature, paired with the present results, suggest that activation of superior parietal areas, such as the paracentral lobule, appears to be more commonly linked to object localization (Haxby et al., 1991; Rauschecker and Tian, 2000; Maeder et al., 2001; Reed et al., 2005; Renier et al., 2009). The exact implications of Porter and colleagues (Porter et al., 2005) findings therefore remain unclear.

### Comparison between ICA and GLM

An earlier study on chemosensory stimulation with CO_2_ has compared regression-based analysis of fMRI based data using the GLM with that of group analysis using the ICA methods. Some activations were only detected by group ICA, but not by GLM; this could be explained by the fact that activity in these regions was shifted temporally and therefore delayed with respect to the expected response. Furthermore, it showed a variation of CO_2_-stimulus-evoked responses which was different for the selected ROIs within one subject (Schopf et al., 2011). This finding of differing hemodynamic responses across subjects, brain regions and sessions is a known constraint of regression-based methods such as GLM (Aguirre et al., 1998; Cunnington et al., 2003; Neumann et al., 2003; Handwerker et al., 2004; Menz et al., 2006). Fully exploratory analysis methods such as ICA [introduced by (McKeown et al., 1998)] do not require the specification of a model or a hemodynamic response function. A number of approaches have been developed to extend ICA from the analysis of a single data set to the group level (Calhoun et al., 2009); the most widely adopted method is to concatenate single-subject data in time prior to performing ICA (Calhoun et al., 2001; Beckmann and Smith, 2005) [for a comparison of toolboxes using temporal concatenation ICA see (Schopf et al., 2010)].

A challenge in group ICA is the need to identify and evaluate group components. This can either be done by temporally correlating the model time course with the corresponding time courses of the group components or by template matching, which includes the spatial correlation of a predefined template with the group component maps. For the present data, we used temporal correlation to find spatial activity patterns across subjects. As hypothesized earlier (Schopf et al., 2011) our study showed that group ICA provides supplemental information—in our case regarding parallel pathways processing—in addition to a priori defined model-dependent regression-based analysis.

## Conclusion

Earlier studies have demonstrated that cerebral architecture follows a subdivision into two parallel sensory processing pathways linking modality specific primary regions with amodal processing regions (posterior parietal cortex for the dorsal pathway, temporal, and inferior parietal regions for the ventral pathway) to frontal regions where both pathways terminate (Reed et al., 2005). Our study using both exploratory and model-driven methods of fMRI analysis revealed results which fit into this framework and extends it to the chemical senses. Taken together, these data suggests that, as for our sensory modalities, the neural processing of intranasal chemosensory stimuli appears to follow a dual pathway model.

### Conflict of interest statement

The authors declare that the research was conducted in the absence of any commercial or financial relationships that could be construed as a potential conflict of interest.
